# A contemporary approach to developing health policies: the Dubai Health Authority as a case study

**DOI:** 10.3389/fpubh.2026.1864765

**Published:** 2026-07-01

**Authors:** Nahed Monsef, Eldaw Suliman, Yamen Elgeneadi, Elham Ashkar, Amna AlMheiri, Shahd Mohammad Arjomand

**Affiliations:** 1 Strategy & Governance Department, Dubai Health Authority, Dubai, United Arab Emirates; 2 Dubai Medical University, Dubai, United Arab Emirates; 3 Department of Health Policy, London School of Economics and Political Science, London, United Kingdom; 4 University of Sharjah, Sharjah, United Arab Emirates

**Keywords:** Adjusted Priority Index, Dubai Health Authority, health policy, policy framework, theory of change

## Abstract

**Introduction:**

Health governance is shifting from reactive decision-making to proactive, evidence-informed policy processes. In Dubai, the Dubai Health Authority’s repositioning as a regulator and health-system steward created a need for a more systematic approach to health policy prioritization, development, and evaluation.

**Context:**

Health policy development in Dubai was historically strategy-driven and top-down. Although this supported rapid sectoral growth, it also risked fragmented decision-making, limited cross-system coordination, and inconsistent assessment of policy impact. Drawing on evidence-informed policymaking and Kingdon’s multiple streams framework, the Strategy and Governance Department conceptualized policy development as the alignment of policy problems, technically feasible options, and political and stakeholder legitimacy.

**Programmatic elements:**

The methodology uses the Adjusted Priority Index, a semi-quantitative MCDA-informed agenda-setting tool, to prioritize prospective policy research topics according to legal feasibility, strategic alignment, government directives, risk, opportunity, urgency, and expected impact. Prioritized topics are then proceeded through structured policy labs covering problem definition, policy framework development, stakeholder analysis, situational assessment, benchmarking, alternatives analysis, theory of change, implementation planning, and impact assessment. Policy champions, internal guidelines, and recurring workshops support methodological consistency and institutional capacity.

**Discussion:**

Early outputs include a three-year health policy agenda, structured policy rationales, transparent prioritization records, stronger cross-departmental coordination, and improved methodological literacy among policy teams and stakeholders. These outputs indicate institutional uptake and implementation readiness, but not final policy effectiveness.

**Conclusion:**

Dubai’s experience shows how evidence-informed policy methods can be adapted to a complex and rapidly evolving health system. Formal evaluation is needed to assess implementation quality, policy coherence, and longer-term contributions to health-system performance.

## 1 Introduction

The global health governance landscape is undergoing profound transformation, marked by a strategic shift from reactive, *ad hoc* approaches to proactive, evidence-informed, and policy-centric decision-making. This paradigm shift is essential for effectively addressing the multifaceted and dynamic health challenges of the 21st century, including the escalating prevalence of chronic diseases, the emergence of novel infectious threats, and the growing societal demand for value-based, equitable, and sustainable health systems. In this context, the World Health Organization (WHO) and many global health institutes have repeatedly emphasized the foundational role of comprehensive and coherent national health policies in strengthening health system resilience and responsiveness (1).

### 1.1 Dubai health sector landscape

Dubai has emerged as a regional leader in healthcare regulation and innovation. Under Law No. 14 of 2021, which amends Law No. 6 of 2018, the Dubai Health Authority (DHA) was repositioned from a regulator–operator to a regulator with system-stewardship responsibilities (2). Its mandate now includes oversight of healthcare delivery across the public and private sectors, licensing and accreditation, monitoring of public health trends, and enforcement of quality and patient safety standards. The DHA also stewards health expenditure, health data governance, and the institutionalization of evidence-informed practice. This mandate aligns with the Dubai Health Strategy 2026, Dubai Social Agenda 2033, and Executive Council directives (3, 4), creating a favorable environment for systematic policy development and value-based initiatives.

Over the last decade, Dubai’s health sector has evolved substantially through coordinated strategic reform. According to the Health Accounts System of Dubai (HASD) published in 2024, total health expenditure reached approximately USD 6.6 billion, equivalent to 5.5% of the UAE’s gross domestic product (GDP). This represented a 10% increase from USD 6.0 billion in 2023 (5). Public financing accounted for 39% of total spending, approximately USD 2.3 billion, while private sources, including insurance and out-of-pocket payments, contributed 62%. During the same period, Dubai’s health financing landscape changed markedly through the achievement of universal health coverage under Law No. 11 of 2013 concerning Dubai Health Insurance (6), the introduction of unified healthcare policy, diagnosis-related group (DRG) reimbursement, dedicated funds for high-cost oncology services for low-income groups, and a unified drug formulary for insured members enrolled in the Essential Benefit Plan (7).

### 1.2 Approach to health policy development in Dubai

Historically, public policy development in Dubai, including health policy, was guided mainly by strategic plans and implemented through a top-down approach. Although this model supported rapid sectoral growth, it also risked fragmented decision-making, limited coordination across the health system (8, 9), and weaker alignment between resource allocation and changing population needs. It also highlighted the absence of a standardized, agile, evidence-based methodology for priority setting and policy impact evaluation. In response, the Strategy and Governance Department (SGD) in the DHA adopted a more systematic evidence-informed approach to address the complex and interconnected challenges facing Dubai’s health sector. The approach aimed to strengthen transparency, accountability, and value while aligning policy development with global practice and Dubai’s evolving health needs.

### 1.3 Objectives and questions to be answered

This case study addresses three questions: (i) what challenges triggered the reform? (ii) how was the evidence-informed methodology for prioritizing and developing health policies conceptualized and implemented? and (iii) what outcomes and impacts are anticipated? These questions are important because health policymaking is shaped by the interaction of technical, institutional, and political factors. Advancing evidence-informed policymaking (EIPM) requires a clear understanding of the core attributes and contextual conditions that influence the policy process. Translating this understanding into practice remains difficult in both high-income and middle-income settings. This is particularly relevant in the Middle East, where empirical evidence on how policy-relevant findings are translated into actionable health strategies remains limited.

## 2 Context

### 2.1 The complexity of health policy development: evidence from the literature

The WHO Global Evidence to Policy (E2P) summit, held in 2023, reinforced the role of researchers, policy analysts, and decision-makers as partners in advancing evidence-informed policymaking (EIPM) across settings (10). EIPM prioritizes the use of the best available research evidence in policy decisions. It provides a systematic and transparent process for accessing, appraising, and applying evidence to policy analysis and decision-making. Although policymaking is not always systematic or transparent, EIPM employs structured methods to identify, assess, and appropriately use relevant research (11, 12).

EIPM is widely recognized as a foundation for resilient health systems, quality health coverage, and progress toward the United Nations Sustainable Development Goals (13, 14). However, translating this principle into practice remains difficult. Health policymaking is rarely linear. It is shaped by health-system complexity, competing stakeholder interests, and socioeconomic and political contexts that influence whether policies are adopted and implemented effectively. These challenges are evident in low- and middle-income countries, where policy capacity and resources may be constrained, but they are also present in high-income countries with more complex health and social systems (15–17). In the Middle East, where countries vary widely in income, health-system capacity, and equity, empirical research on translating policy-relevant evidence into actionable health strategies remains limited (18, 19). This gap creates a vulnerability for regional health security, system resilience, and development.

### 2.2 Agenda-setting theory for policymaking

Kingdon’s multiple streams framework, also known as agenda-setting theory, conceptualizes policymaking as the interaction of three semi-autonomous streams: problems, policies, and politics (20, 21). The problem stream refers to how societal conditions are identified, framed, and legitimized as requiring government action. The policy stream concerns the development and refinement of possible policy alternatives. The political stream captures the institutional, ideological, and stakeholder influences that shape the decision-making environment, both inside and outside formal government structures (22). For a health policy to be adopted and implemented as a public directive, scientific evidence must be combined with political legitimacy and administrative feasibility (23).

### 2.3 Adopting a scientific approach

Drawing on Kingdon’s theory, the SGD conceptualized health policy prioritization and development as the deliberate alignment of the problem, policy, and political streams. This framing aimed to ensure that proposed reforms were technically sound, politically feasible, and socially legitimate. Within the problem stream, DHA had already developed a strong analytic capacity through health registries and administrative data, which helped characterize pressing health challenges such as the rising burden of non-communicable diseases and variation in health and well-being indicators. The SGD, together with relevant DHA organizational units, extended this function beyond data surveillance by translating epidemiological and health-economic evidence into clearly defined policy problems. These were then expressed as policy rationales that could be understood by policymakers, stakeholders, and the public.

Within the policy stream, the SGD used its partnerships with ranked academic institutions, think tanks, and professional associations (24, 25) to develop a structured three-year health policy agenda. This agenda represents a planned list of policy research topics for health reform in Dubai. Proposed topics are assessed against criteria including mandate fulfillment, legal and strategic alignment, government directives, urgency, risk, opportunity, and expected impact. This assessment helps ensure that policy topics are both evidence-informed and aligned with Dubai’s health-sector priorities, including the Dubai Health Strategy 2026, Dubai Social Agenda 2033, and TEC directives.

The political stream requires attention to legitimacy, feasibility, and stakeholder support. In Dubai, this involves engaging stakeholders across government and the private sector, aligning policy priorities with broader social and economic goals, and securing the institutional support needed for adoption. The SGD methodology operationalizes this process as a cyclical model in which policy problems, potential solutions, and political alignment are reviewed and updated. By combining evidence, policy design, and structured deliberation, the approach aims to strengthen confidence in the objectivity and transparency of policy decisions (26).

The DHA methodology was developed through an iterative review of international practice in evidence-informed policy development, particularly guidance from the WHO and the UK’s National Institute for Health and Care Excellence (NICE) (27). Principles from these sources were adapted to Dubai’s governance context. Central elements include a transparent process for identifying and prioritizing policy topics, structured policy analysis, and systematic stakeholder engagement. The process also requires developing a policy framework that defines the scope, pillars, sub-pillars, and enablers for each policy area (28). These elements guide the situational analysis, stakeholder-informed assessment, benchmarking, policy alternatives, and final policy direction. Stakeholder engagement and high-level political endorsement are treated as necessary components of policy development, ensuring that policy priorities are justified by scientific evidence while remaining responsive to institutional mandates and stakeholder expectations.

## 3 Programmatic elements

### 3.1 The Adjusted Priority Index of health policies in Dubai

In the DHA’s pursuit of EIPM in the health sector, the SGD developed the Adjusted Priority Index (API), a semi-quantitative, MCDA-informed tool for prioritizing health policy research topics. The API guides policy agenda-setting by indicating whether prospective policy topics should be added to DHA’s three-year health policy research plan, their urgency, and the sequencing of policy research resources. It does not determine final policy options or interventions. Once a topic is prioritized, the assumptions underlying the API score are tested and refined through situational analysis, current-state assessment, stakeholder evidence review, benchmarking, alternatives analysis, feasibility assessment, theory-of-change development, and final approval.

**Figure 1:**
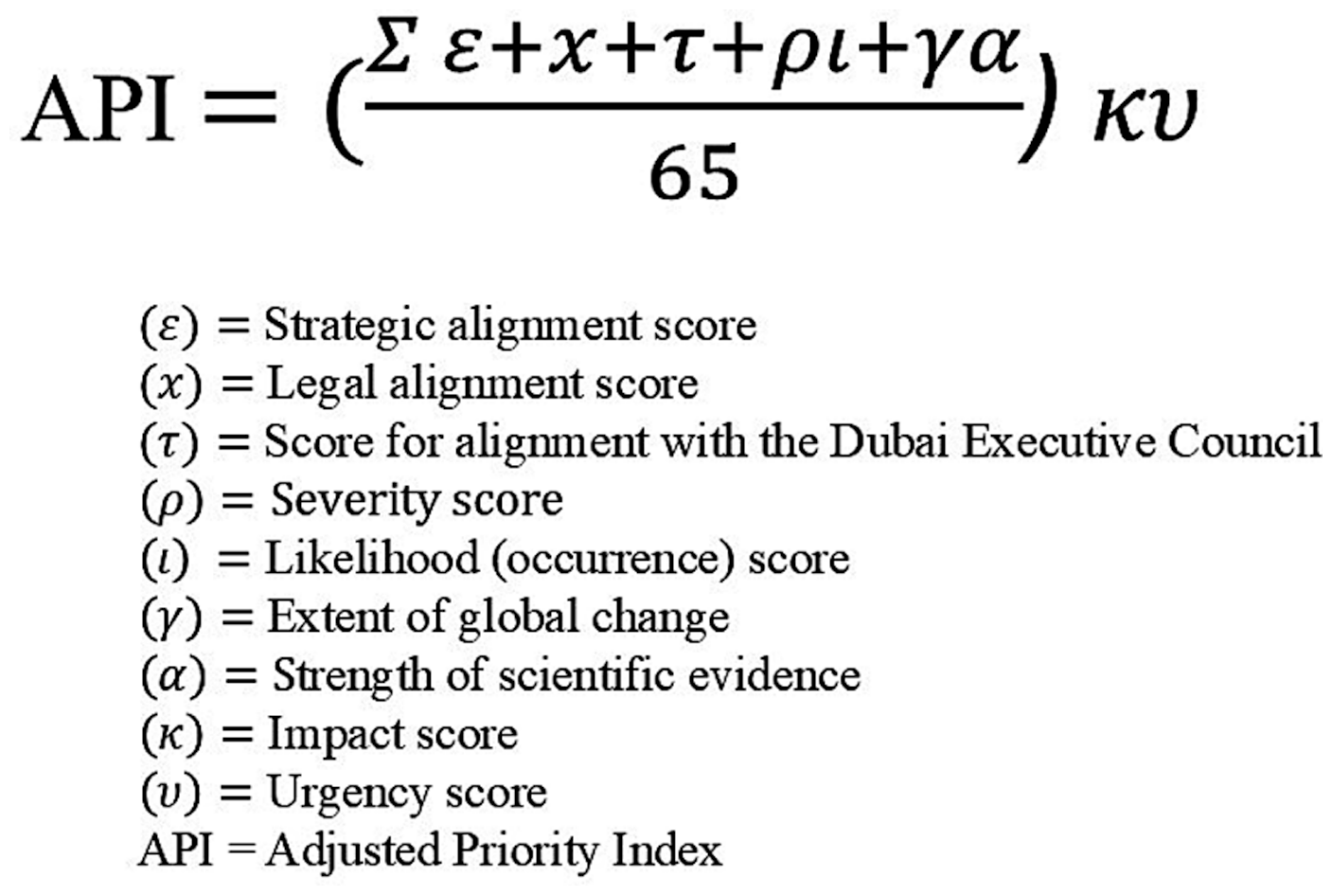
Adjusted Priority Index formula and scoring structure – presentation of the API formula and the criteria used to generate the adjusted prioritization score; detailed scoring definitions are provided in Appendix 1.

The API evaluates each topic using a predefined five-point rubric covering legal alignment (x), strategic alignment (*ε*), alignment with governmental directives represented by TEC priorities (*τ*), risk ( ρι) , opportunity in change ( γα ), impact (*κ*), and urgency (*υ*). Risk is calculated as severity (ρ) multiplied by likelihood of occurrence (ι), using the risk matrix approach (29, 30). Opportunity in change combines the extent of assumed global, regional, or local change (*γ*) with the strength of supporting evidence (*α*), drawing on evidence hierarchies such as the Oxford Center for Evidence-Based Medicine levels of evidence (31, 32) and GRADE (33, 34). The base priority score follows the Eisenhower matrix of impact multiplied by urgency (35, 36), and then applies contextual adjustment criteria to generate the final API score (Figure 1; Appendix 1).

The API uses a fixed aggregation structure rather than empirically derived preference weights. The contextual adjustment factor is calculated as [*ε* + x + *τ* + (*ρ* × *ι*) + (*γ* × *α*)] / 65, where 65 is the maximum possible contextual score; the final API is calculated as API = [(ε + x + τ + ρ × ι + γ × α) / 65] × (*κ* × *υ*). Risk and opportunity, therefore, have greater relative influence because they are compound criteria, whereas impact and urgency remain the core drivers of priority. The weighting structure was selected through internal methodological deliberation and adaptation to DHA’s legal, strategic, and governance context; therefore, the API should be interpreted as a transparent decision-support and prioritization tool, rather than as a fully externally validated preference-weighting instrument.

The initial API development did not use a formal Delphi process or nominal group technique. Instead, API scores are aggregated through a structured internal expert-informed deliberative process involving the SGD, relevant DHA organizational units, policy champions, and subject-matter stakeholders. During application, each score must be justified in an API card that records the qualitative rating, numerical score, evidence source, and rationale for each criterion as established in Appendix 1. Scoring differences are resolved through a moderated review of the scoring definitions and available evidence. These procedures reduce, but do not eliminate, subjective judgment; they support internal face and content validity, while formal external validation, statistical inter-rater reliability assessment, and sensitivity analysis remain priorities for future validation.

### 3.2 Methodology for health policy development in Dubai

**Figure 2:**
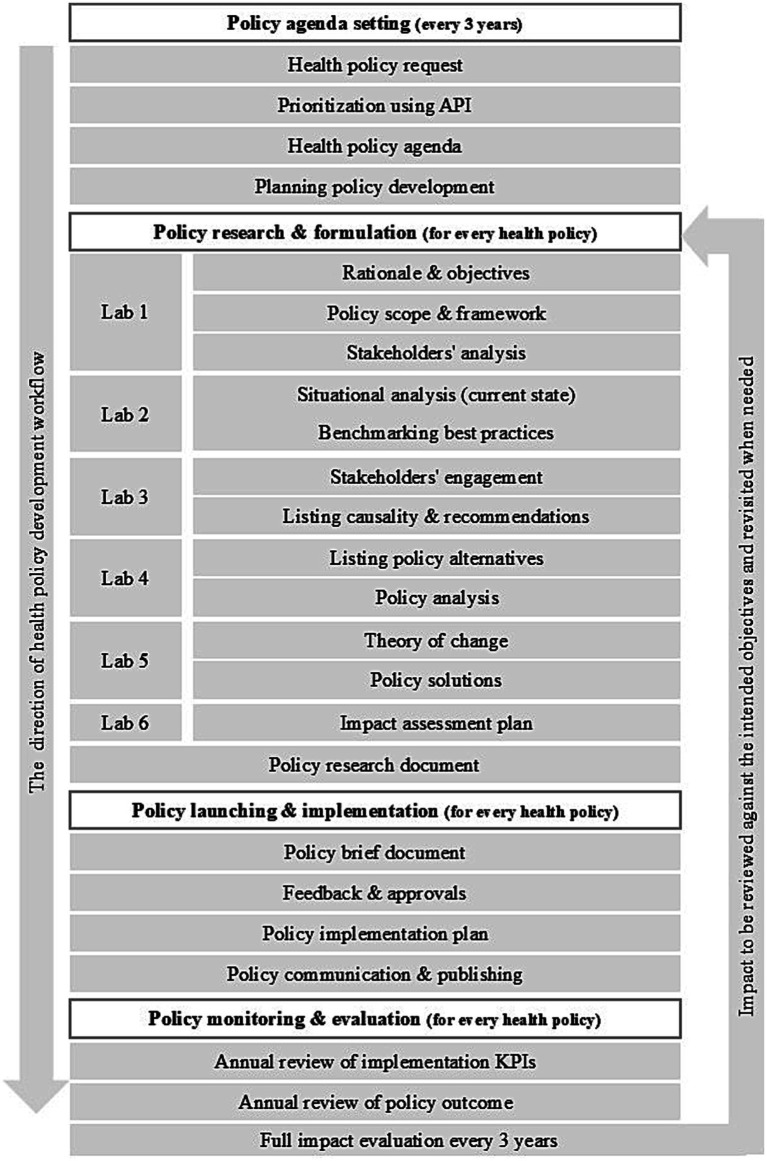
Complete DHA health policy development cycle—summary of the pathway from policy request and API prioritization to policy research, approval, publication, KPI monitoring, and future evaluation.

Following API-based prioritization, topic master planning is assigned to the SGD team and the relevant organizational unit to begin policy research in accordance with the DHA health policy development cycle (Figure 2). The API provides the entry point for agenda-setting sequencing; the subsequent policy labs translate the prioritized topic into a defined policy problem, scope, evidence base, alternatives, preferred interventions, theory of change, implementation plan, and evaluation approach.

Policy development begins by refining the policy rationale submitted through the policy request form, defining the scope, and developing the policy framework. The framework is decomposed into pillars, sub-pillars, enablers, and topic-specific activities that structure the current-state assessment, benchmarking, gap analysis, causality mapping, recommendations, and intervention design (Appendix 2). Stakeholder engagement is operationalized early through a semi-qualitative power-interest analysis: stakeholders are listed according to their responsibility, accountability, influence, or affected status, and anonymous power scores are combined with interest counts to generate an influence map that determines whether stakeholders receive sustained engagement, targeted consultation, or information updates during development and launch (Appendix 3).

**Figure 3:**
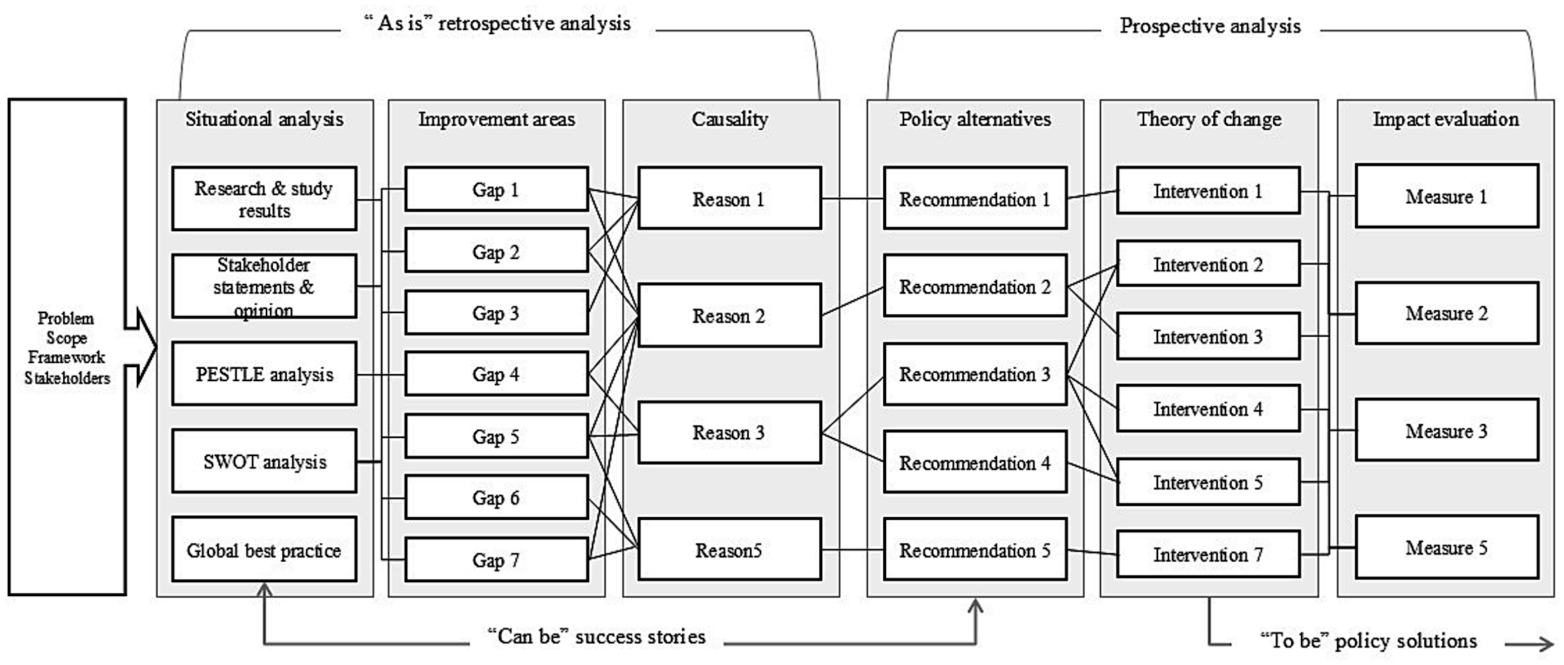
Flow of information during health policy formulation and policy lab work – explanation of how current-state evidence, stakeholder input, benchmarking, gap analysis, recommendations, alternatives, theory of change, and impact measures are linked during policy formulation.

The situational analysis evaluates health determinants and needs, service demand, system performance, sector capacity, available resources, and identified gaps. Benchmarking of regional and international practice is then used to identify feasible recommendations and policy alternatives. Current-state findings are synthesized through gap and causality mapping, linking observed gaps to their most clearly articulated causes and recommended policy alternatives, as illustrated in the flow of information during policy formulation (Figure 3). Recommendations are then examined through policy analysis, including alternatives assessment, impact simulation, effectiveness appraisal, and feasibility assessment, where data allow. Stakeholder engagement sessions are used to test the interpretation of evidence and improve the relevance and feasibility of recommendations.

The transformation of recommendations into actionable interventions is guided by quantitative analysis or qualitative assessment with influential stakeholders. The responsible organizational unit then proposes a theory of change for each selected intervention, presenting the required inputs, anticipated outputs, expected outcomes, projected impacts, supporting evidence, and assumptions to decision-makers (Appendix 4). Following political endorsement, policy statements are drafted and published on the DHA digital platform. Each approved policy is supported by an implementation plan with assigned responsibilities, translated into departmental KPIs, monitored by DHA’s Quality and Performance function, and reviewed during annual internal audits as an additional governance check. An impact-assessment framework is also established to support ongoing evaluation, and policy modification requires a subsequent policy study that demonstrates alternative impacts and secures political approval.

### 3.3 Capacity building for health policy development labs

The institutionalization of a methodology to prioritize and develop an evidence-based health policy for the Dubai health sector required deliberate investments in capacity building and skills development among the internal teams responsible for policy design. Effective prioritization processes necessitate not only technical competencies in health economics, public health, and policy analysis, but also transversal skills in stakeholder engagement, improvement processes, and systems thinking. To implement this approach, each organizational unit within the authority nominated a “policy champion” responsible for ensuring that subject-matter expertise was applied effectively to the relevant policy domains and for serving as a liaison to maintain horizontal communication across departments throughout the research and policy process. This distributed leadership model helped mitigate the risk of fragmentation by embedding accountability and technical insight at the operational level, thereby fostering alignment between specialized expertise and broader governmental and institutional guidelines such as the *Public Policy Development Guidebook* by the United Arab Emirates (UAE) Government (37), and the *Public Policy Guide of the Government of Dubai* published in 2018 by The General Secretariat of TEC (38).

**Figure 4:**
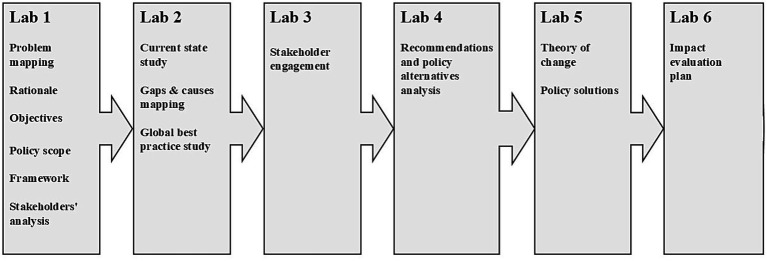
DHA health policy research and formulation labs—brief of the six policy labs used to move from problem mapping and scope definition to stakeholder engagement, theory of change, policy solutions, and impact-evaluation planning.

To further consolidate methodological coherence, the SGD introduced health policy development guidelines detailing the policy labs. These have gained global recognition as innovative spaces for co-design and rapid prototyping of solutions. They provide a structured environment in which policymakers, researchers, and policy stakeholders collaboratively test, refine, and adapt policy instruments prior to full-scale implementation (39). The policy development labs are a combination of interviews, brainstorming, and focus group sessions that aim to first train the stakeholders involved in the use of the tools of each developmental stage, and second, to conduct semi-qualitative analysis and open discussions on the health policy topic within the context of the lab, whether a prospective analysis or retrospective studies (40). The SGD has developed a series of six structured policy labs to capture and channel information flows across both retrospective studies and prospective policy analyses (Figure 3). These labs are organized sequentially to reflect logical progression from defining the problem to choosing a solution (Figure 4).

Lab 1 focuses on articulating the rationale and objectives underpinning the policy initiative, whereas. It also clarifies the policy scope and framework and incorporates a stakeholder power analysis to identify the most influential actors to engage with throughout policy development. Lab 2 facilitates comprehensive discussions on the current state of the health system, complemented by a review of global and regional best practices. In Lab 3, the identified stakeholders convene to examine the evidence, map existing gaps, analyze causality, and formulate preliminary recommendations through essential policy stakeholder engagement roundtable(s). While Lab 4 is dedicated to the systematic analysis of recommendations and suggested policy alternatives, the process culminates in Labs 5 and 6, which conclude the formulation of proposed interventions using the theory of change model, the selection of policy solutions, and the development of impact assessment measures. Alongside lab training, the DHA holds semi-annual mandatory workshops for policy champions and teams to reinforce its policy development methodology. These workshops encourage iterative learning and ensure staff are proficient in using the prioritization framework and tools. External seminars with academic partners supplement internal training by sharing progress and lessons learned. Together, these initiatives foster adaptive learning and maintain robust, sustainable policy processes within the authority.

## 4 Discussion

### 4.1 Immediate outcomes

Following the incorporation of these formulations and methodologies, DHA observed a more structured and systematic approach to agenda setting and agenda review. The semiquantitative approach engaged both policy actors who work primarily with qualitative evidence, including regulation, strategy, governance, and risk management, and technical teams with quantitative expertise in epidemiology, health economics, and data management. This multidisciplinary input broadened the evidence base used during policy formulation and improved the traceability of how recommendations and alternatives were developed. Applying the API established an operational mechanism for appraising competing policy topics and translating them into a structured three-year health policy research agenda. Its influence was therefore observed at the level of agenda-setting, topic inclusion, sequencing, and prioritization timelines, rather than at the level of selecting final policy solutions. Once a topic was added to the agenda, its initial API assumptions were tested through policy research, situational analysis, benchmarking, alternatives analysis, stakeholder deliberation, theory-of-change development, and final approval. This distinction is important because the API provides a transparent starting point for prioritization, while the policy labs and subsequent decision-making processes determine the policy alternatives and interventions.

This process standardized decision-making and strengthened institutional accountability by ensuring that prioritization decisions were documented, traceable, and evidence-informed within a defined level of uncertainty (26, 41, 42). The use of policy champions within each organizational unit further strengthened internal governance, as these designated individuals facilitated cross-departmental communication, safeguarded the technical integrity of inputs, and improved coordination across policy streams within DHA and with external stakeholders. The integration of policy labs and training workshops contributed to shared methodological literacy, ensuring that stakeholders engaged in policy development had both the technical tools and contextual understanding required to apply evidence rigorously. This iterative learning environment has already yielded tangible outputs, including a three-year health policy agenda and the refinement of policy rationales into structured, accessible formats for policymakers.

To strengthen the empirical reporting of these immediate outcomes, DHA mapped the early products of the methodology to measurable implementation and process indicators, including API-screened topics, topics included in the three-year agenda, priority classifications, structured policy rationales, policy labs, stakeholder engagement, policy champions, completed policy frameworks, situational analyses, theory-of-change models, implementation plans, and approved or launched policies (Table 1). These measures should be interpreted as evidence of institutional uptake, implementation readiness, and methodological consistency, rather than as evidence that the resulting policies have already improved health-system performance. Longer-term assessment of policy outcomes and impacts will require post-implementation evaluation against the policy-specific indicators and targets defined through the theory-of-change process.

**Table 1:** Early measurable outputs of the DHA health policy-development methodology (reported as of May 2026).

Outcome domain	Measurable indicators API process started in May 2025 Policy development started in January 2026; outputs reported to May 2026.	Output*	Data source
API screening	Number of prospective policy topics screened using the API	47	API scoring sheets/policy agenda records/API policy cards
Agenda-setting	Number of policy research topics included in the three-year agenda	11	Three-year policy agenda (final list)
Prioritization distribution	Number of topics classified as Crucial,	3	API policy cards/Consolidated master list
	High Priority (added to 3-year policy agenda)	1	
	Medium Priority (added to 3-year policy agenda)	3	
	Low Priority (added to 3-year policy agenda)	4	
	Postponed/Excluded from policy agenda	36	
Policy briefs	Number of completed policy briefs	7	Policy development progress tracking sheet
Policy labs completion	Number of policy labs conducted	42	Lab records/policy development progress tracking sheet
Meetings per policy lab	Average number of meetings/workshops conducted per policy lab	1.28	Lab records/meeting minutes
Completion of lab cycle	Number and percentage of policy topics completing Labs 1–6 (full lab cycle)	7/7100%	Policy development progress tracking sheet
Stakeholder engagement	Number of stakeholder engagement sessions conducted	9	Stakeholders’ engagement: meeting attendance sheets
	Average of participation in every session	7.2	
	Total Number of participants	68	
Policy champions	Number of policy champions nominated	8	Training records
	Number of attendees of policy methodology workshops	52	
Capacity-building	Number of methodology workshops delivered (total of annual trainings and the policy-related workshops)	10	Workshop attendance/evaluations
	Number of trained employees for the methodology	113	
Policy frameworks	Number of policy frameworks developed	7	Framework documents
Situational analyses	Number of current-state assessments and benchmarking reports completed	14	Policy research files
Recommendations and alternatives	Number of recommendations	96	Policy lab outputs
	Number of policy alternatives generated and appraised	56	
Theory of change	Number of policies with ToC, indicators, targets, and assumptions documented	7	ToC templates
Implementation readiness	Number of policies with implementation plans and assigned responsibilities	7	Implementation plans
Policy output	Number of policies approved	7	Approval records/digital platform
	Number of policies published on the DHA digital platform	6	
	Number of policies to be published	1	
Early monitoring	Number of policies with baseline indicators and planned review date	7	Implementation and evaluation plan

*These are reported annually to and by the DHA’s Strategy & Governance Department.

### 4.2 Testing the model

Given that the API is a tool for setting the three-year policy agenda of the Dubai health sector, the first API was piloted through the first policy developed and launched under the DHA policy-development model, namely the health policy on communicable diseases. The initial API score was not treated as a final or self-validating decision; rather, it served as a provisional prioritization signal that was subsequently tested through staged operational reliability tests throughout the policy-development cycle. Risk, opportunity, evidence strength, and problem-definition assumptions are examined in every policy research phase, particularly through situational analysis, current-state assessment, stakeholder evidence review, and benchmarking of regional and international practice. Strategic, legal, and Executive Council alignment scores are re-examined during the final policy approval phase before publication and launch. Impact scores are to be tested during policy alternatives analysis, including feasibility assessment, simulation of policy benefits, effectiveness appraisal, and economic or risk analysis, where data allows (Table 2). This staged process allowed the initial API assumptions to be confirmed, refined, or qualified before final policy approval. It provides an embedded operational reliability mechanism within the DHA policy-development process, while recognizing that formal statistical inter-rater reliability testing using independent scorers has not yet been completed and remains a future validation priority.

**Table 2:** Staged verification checkpoints for API components during policy formulation.

API component	Reliability or verification checkpoint	Evidence source
Risk (severity and likelihood)	Tested during situational analysis, current-state assessment, and review of epidemiological, service, operational, and risk data	Current-state assessment, administrative data, registries, stakeholder evidence
Opportunity (evidence strength, and extent of change)	Tested through benchmarking of global, regional, federal, and Dubai-level practice. Reassessed during evidence review and policy research	Benchmarking report, global best practices, external policy examples Literature review, technical reports, economic evidence, and grey literature
Strategic alignment	Re-examined during policy framework development and final approval	Dubai Health Strategy, Dubai Social Agenda, DHA priorities
Legal alignment	Rechecked during legal and governance review before launch	Laws, decrees, regulatory mandates, and legal review
TEC and political alignment	Tested during final approval and endorsement before publication	Executive Council priorities, senior decision-maker review
Impact	Tested during alternatives analysis, feasibility assessment, benefit simulation, and theory-of-change development	Simulations, economic analysis, risk assessment, stakeholder appraisal
Urgency	Reassessed against policy-development timeline and implementation readiness	Annual plan, organizational request, launch timeline

Since no policy has yet gone through a complete implementation and outcome assessment, initial performance is evaluated using governance and accountability metrics rather than final health-system results. After approval, implementation plans are converted into departmental KPIs, which are assigned to the responsible organizational units; these KPIs are overseen by DHA’s Quality and Performance division, which operates independently of the implementation units. Moreover, the annual internal audit provides a governance-level review, ensuring compliance with the policy development process, approved plans, and designated implementation responsibilities. At the time of reporting, the methodology had supported the development of seven health policies on the following topics: non-communicable diseases, communicable diseases, promoting healthy food, smoking, school health, health coverage for patients with cancer, and health data governance. At the time of reporting, no formal outcome or impact evaluation of the methodology or of the policies developed through it had yet been completed; the evidence presented in this case study should therefore be interpreted as early implementation outputs and governance-based performance monitoring, with formal evaluation planned through policy-specific theory-of-change indicators, departmental KPIs, internal audit, and post-implementation impact assessment.

### 4.3 Anticipated long-term impact

Over the long term, the incorporation of this methodology is anticipated to yield transformative impacts on health sector governance and performance in Dubai. First, the methodology is expected to enhance policy coherence by reducing duplication and mitigating the fragmentation that often arises when health policies are developed in silos. Second, by embedding evidence appraisal and stakeholder engagement as core features of the process, the DHA is likely to promote a stronger culture of data-driven decision-making, thereby reinforcing institutional resilience and adaptability in the face of emerging health threats. Third, the iterative nature of the methodology, particularly through policy labs and recurrent training, ensures a feedback-oriented system that promotes continuous learning and adaptive implementation. Finally, the cumulative policy impact assessment plan, including agreed-upon timely measures and targets, is used to conduct a comprehensive impact analysis of the methodology itself, as the success of policy development and its implementation collectively reflects cohesive improvement in the whole health system’s performance. Nevertheless, by adopting an EIPM approach, Dubai can align its health sector reforms not only with local strategic priorities but also with broader global sustainability frameworks. Ultimately, as per Rehfuess and Best (43, 44), the anticipated impact is the maturation of a health policy ecosystem that is simultaneously responsive, anticipatory, and sustainable, providing a replicable model for other health systems where EIPM may remain underdeveloped.

### 4.4 Limitations and risks

This case study should be interpreted within Dubai’s specific governance context. DHA operates within a centralized health-system stewardship model, with a clear regulatory mandate, access to administrative data, and alignment with government priorities. These conditions supported structured agenda-setting and policy development, but they may limit the direct transferability to decentralized health systems or lower-resource settings with different legal mandates, weaker data systems, or less capacity for policy analysis.

The methodology was developed and initially applied within DHA, which may introduce institutional bias. The API was not developed through a formal Delphi process, nominal group technique, or independent external expert panel, and it has not yet undergone external validation, formal statistical inter-rater reliability testing, or sensitivity analysis. Although predefined scoring rubrics, API cards, evidence documentation, stakeholder review, and staged verification reduce subjectivity, some criteria, particularly strategic alignment, Executive Council alignment, urgency, and anticipated impact, remain partly shaped by institutional and political interpretation. These factors are relevant to the feasibility of public policy, but they may also influence scoring and sequencing decisions.

Finally, the methodology is resource-intensive and requires policy researchers, technical experts, policy champions, stakeholder time, data access, facilitation capacity, and governance follow-up. The evidence reported in this case study reflects early implementation outputs and governance-based performance monitoring rather than completed outcome or impact evaluation. Therefore, claims about effectiveness, health-system improvement, quality, safety, equity, access, cost, or population-health impact remain premature. Future work should include external validation of the API, inter-rater reliability testing, sensitivity analysis, assessment of implementation fidelity, and post-implementation evaluation using the indicators and targets defined through the theory-of-change process.

## 5 Conclusion

This case study illustrates DHA’s shift from a predominantly strategy-driven model to a more structured, evidence-informed policy process. Through the API, policy frameworks, stakeholder engagement, policy labs, theory of change, implementation planning, and policy champions, DHA has institutionalized a transparent and accountable approach to health policy prioritization and development. The initial results reported in this case study, such as the three-year health policy agenda, completed policy briefs, organized policy labs, theory-of-change models, implementation plans, and approved policies, indicate that the methodology has been adopted operationally and has enhanced the traceability of policy decisions. Nevertheless, these results should be viewed as evidence of institutional adoption and methodological consistency, rather than proof of the policies’ final effectiveness.

Future efforts should move from developing the methodology and conducting initial monitoring to performing formal evaluations. This includes validating external APIs, testing inter-rater reliability, performing sensitivity analyses of scoring assumptions, and conducting prospective fidelity checks using departmental KPIs, Quality and Performance metrics, and internal audits. Once implemented, evaluations should assess whether policies derived from this process lead to measurable improvements in policy coherence, implementation quality, access, safety, equity, costs, population health, and overall health system performance. Applying this approach in other UAE, Gulf, or similar governance contexts will help identify which elements are generally transferable, which require modification, and whether the DHA model can support evidence-based policymaking beyond its original setting. Future methodological research could compare the DHA model with other international prioritization and evidence-to-decision frameworks to assess its transferability beyond Dubai’s governance environment.

## Data Availability

The original contributions presented in the study are included in the article/Supplementary material, further inquiries can be directed to the corresponding authors.
