# Supplementary material for: A contemporary approach to developing health policies: the Dubai Health Authority as a case study
Source: Front Public Health. 2026 Jul 1;14:1864765. doi: 10.3389/fpubh.2026.1864765 (PMC13369604; doi:10.3389/fpubh.2026.1864765)
Supplement: Supplementary file 1 [file Data_Sheet_1.pdf]

Appendix 1. Adjusted Priority Index: A tailored methodology for prioritizing health policies

Background

The global health landscape is evolving towards proactive, evidence-based policy making to tackle complex health issues of the 21st century. In this environment, Dubai's health sector, primarily driven by top-down strategic planning, encountered challenges such as system fragmentation and inefficient resource use. This led the Dubai Health Authority (DHA) to see the necessity of shifting from a traditional, directive approach to a more evidence-based, policy-driven governance model. The DHA's Strategy and Governance Department (SGD) has established and applied a structured, scientific process for ranking health policy research priorities based on emerging risks and global changes. This document offers a detailed analysis of this index, culminating in the creation of a well-defined five-point scale ratio for each dimension. Supported by academic research from reputable institutions and including operational definitions, the framework aims to facilitate practical implementation.

Rationale

Effective prioritization is critical to organizational performance, particularly in contexts where resources are constrained and competing demands necessitate strategic allocation of time and effort. While numerous frameworks exist, the most widely adopted approaches assess tasks along two primary dimensions: impact and feasibility, or impact and urgency. However, these two dimensions do not carry any tailored weights or corporate alignment to suggest accuracy and relevancy to local context [1,2]. Following consultations with the DHA's SGD, it was noted that additional dimensions have been employed to refine the prioritization matrix. Among these, feasibility was previously considered but has since been excluded from the core prioritization equation. Instead, all dimensions recommended by the SGD have been integrated as adjustment factors contributing to a comprehensive priority index. This document introduces a scientifically informed prioritization framework using a multiple-criteria decision analysis (MCDA) system for the Dubai health sector, to support strategic and political initiatives, such as projects and policies. The weighting criteria used to adjust the analysis are as follows:

a) The legal complementation of the suggested policy or project
b) The strategic alignment with the Dubai health sector strategy 2026 and the Dubai social agenda 33
c) The government directives represented in the priorities of the Executive Council (TEC) of the Dubai Government
d) The magnitude of risk in imaging health issues
e) The opportunity in global trends and change

The MCDA is structured around a five-level Likert scale and grounded in extensive academic research. Following a comprehensive evaluation of various tools, the impact–urgency matrix—commonly referred to as the Eisenhower Matrix [3,4]—was identified as the most robust and academically endorsed model. This framework traditionally calculates priority by multiplying impact scores with feasibility scores, thereby offering a balanced assessment that accounts for both the strategic value of tasks and the practical constraints of execution.

Calculation method

$$\text{API} = \left(\frac{\Sigma\ \varepsilon + x + \tau + \rho\iota + \gamma\alpha}{65}\right) \kappa\upsilon$$

(ε) = Strategic alignment score
(x) = Legal complementation score
(τ) = Score for alignment with the Dubai Executive Council
(ρ) = Severity score
(ι) = Likelihood (occurrence) score
(γ) = Extent of global change
(α) = Strength of scientific evidence
(κ) = Impact score
(υ) = Urgency score
API = Adjusted Priority Index

Figure A1.1. The formula used to calculate the Adjusted Priority Index.

In DHA's pursuit of an EIPM in health sector, SGD has tailored a sophisticated, semiquantitative methodology for the prioritization of health policies, termed the API. This index is anchored in a MCDA framework, a robust approach that facilitates the systematic evaluation of prospective policy topics against a comprehensive suite of criteria. Beyond the conventional, yet critical, considerations of legal complementation (x) (Table 1), strategic alignment (ε) (Table 2), and governmental directives (τ) (Table 3), the API integrates a more nuanced set of variables. A key component is a scientifically derived risk score, which is calculated using the risk matrix approach (RMA) (Table 6,7) [5,6]. This involves quantifying the burden of disease and emerging health threats by multiplying the severity of the health issue (ρ) (Table 4) by its likelihood of occurrence (ι) (Table 5). Concurrently, the index evaluates the "Opportunity for change", (Tables 10,11) a metric derived from the extent of change and trends (γ) (Table 9) and the quality of available scientific evidence (α) (Table 8). The strength of this evidence is meticulously graded on a five-point scale, drawing upon established hierarchies such as the Oxford Centre for Evidence-Based Medicine (CEBM) levels of evidence [7,8] and the Grading of Recommendations Assessment, Development and Evaluation (GRADE) system for rating evidence quality in clinical practice guidelines [9,10]. This ensures that the evidence underpinning any potential policy is rigorously assessed for its validity and applicability. These multi-faceted criteria collectively form a weighting factor to adjust the classic Eisenhower priority score (Tables 14, 15), a product of a policy's impact (κ) (Table 12) and urgency (υ) (Table 13), to yield the final API score (Table 16 and Figure 1).

The API was designed as a semi-quantitative, MCDA-informed prioritization index with a fixed aggregation structure rather than an empirically estimated preference-weighting model. Each prospective policy topic is scored against predefined five-point anchors for strategic alignment (ε), legal alignment (x), alignment with The Executive Council priorities (τ), severity of the health or system risk (ρ), likelihood of occurrence (ι), extent of global, regional, or local policy change (γ), strength of supporting evidence (α), expected policy impact (κ), and urgency of policy development (υ). The base priority score is calculated as expected impact multiplied by urgency (κ × υ). This base score is then adjusted by a contextual suitability factor composed of three single-domain alignment criteria and two compound criteria: risk, calculated as severity multiplied by likelihood (ρ × ι), and opportunity for change, calculated as extent of change multiplied by strength of evidence (γ × α). The numerator of the adjustment factor is therefore ε + x + τ + (ρ × ι) + (γ × α). Because the maximum possible values for these components are 5, 5, 5, 25, and 25, respectively, the denominator is fixed at 65, converting the adjustment factor into a proportion of the maximum possible contextual score. The final API is calculated as: API = [(ε + x + τ + ρ × ι + γ × α) / 65] × (κ × υ). This structure gives greater relative influence on risk and opportunity because both are treated as compound constructs, while preserving impact and urgency as the core priority-driver. Scores are assigned using predefined rubrics and are accompanied by qualitative justification and supporting evidence for each criterion. The weighting structure was selected through internal methodological deliberation and adaptation to DHA's legal, strategic, and governance context; therefore, the API should be interpreted as a transparent decision-support and prioritization tool, rather than as a fully externally validated preference-weighting instrument

All the aforementioned factors in the MCDA are based on a defined five-point score system, which is evaluated through in-depth discussion with relevant stakeholders and primary research on positive and negative trends. The initial development of the API did not use a formal Delphi process or nominal group technique. Rather, the API scores were concluded through a structured internal expert-informed deliberative process (policy agenda brainstorming workshops) led by the Strategy and Governance Department, with input from relevant DHA organizational units and policy champions. During application, policy topics are scored using predefined five-point rubrics, evidence summaries, and stakeholder discussion; scoring differences are resolved through moderated review of the scoring definitions and available evidence. Final scores are documented in an API card that records the qualitative rating, numerical score, supporting evidence, and rationale for each criterion. This approach supports internal face and content validity but does not constitute formal external consensus validation and inter-rater reliability assessment are planned as future validation steps. Accordingly, this composite index provides a nuanced, evidence-based assessment of the criticality of developing a given health policy at a specific point in time. Fundamentally, the API serves as a crucial preliminary assessment tool. It is designed to structure, inform and be tested by the pre-policy analysis phase, fostering a scientifically grounded dialogue, both qualitative and quantitative, among stakeholders before significant resources are committed to full-scale policy development

Level & score definitions for the multiple criteria decision analysis (MCDA)

Table A1.1. Definitions of the legal complementation (x) levels.

Level	Score	Description
Complete Alignment	5	The policy exceeds legal requirements, anticipates legal developments, and represents best practice in legal compliance.
Adequate Alignment	4	The policy comprehensively addresses specific law, administrative decree, local order or legal requirements and aligns closely with current legislation - The policy anticipates common compliance challenges and provides solutions.

Moderate Alignment	3	The policy meets essential legal requirements but may not address all relevant legislation, or amendments or may lack comprehensive coverage of all legal nuances
Limited Alignment	2	The policy meets only the most basic legal requirements, with significant gaps or areas of vulnerability, debate and/or ethical arguments - The policy contains ambiguous language that could lead to non-compliant implementation - The policy meets the letter of the law in some areas but misses the intent - The policy lacks sufficient detail on legal requirements to ensure proper implementation
Not Aligned	1	The policy contains elements that may directly conflict with existing laws or regulations or fail to address mandatory legal requirements in its domain

Table A1.2. Definitions of the strategic alignment (ε) levels.

Level	Score	Description
Complete Alignment (Strategic Theme /Priority)	5	The policy / mission covers a complete theme or strategic priority with and essential to the organization's core strategic approach - outcomes are directly measured as strategic key performance indicators
Strong Alignment (Strategic Objective)	4	The policy / mission covers a clear strategic objective under specific priority - outcomes are directly measured as strategic key performance indicators
Integrated Alignment (Strategic Initiative)	3	The policy / mission has clear connections to some strategic Initiatives but may not address a full core strategic priorities or objectives - outcomes are indirectly measured as strategic key performance indicators
Limited Alignment	2	The policy / mission has tangential or indirect connections to the organization's strategic objectives - outcomes are indirectly measured as strategic key performance indicators
Not Aligned	1	The policy / mission has little to no connection with the organization's strategic objectives. - outcomes are not measured as strategic key performance indicators

Table A1.3. Definitions of the governmental directives (τ) and alignment with The Executive Council (TEC) priorities in Dubai - levels' definitions

Level	Score	Description
Complete Alignment	5	The policy is fundamentally integrated with Dubai Executive Council directives and is essential to achieving the Emirate's strategic vision.
Adequate Alignment	4	The policy has substantial connections to important Dubai Executive Council directives and clearly advances the Emirate's strategic agenda.
Moderate Alignment	3	The policy has clear connections to some Dubai Executive Council directives but may not address core strategic priorities.
Limited Alignment	2	The policy has tangential or indirect connections to Dubai Executive Council directives with minimal strategic relevance.
Not Aligned	1	The policy is isolated from the Dubai Executive Council directives and may contradict the Emirate's strategic priorities.

Table A1.4. The likelihood of risk occurrence (ι) levels' definitions.

Level	Score	Description
Certain	5	Expected to occur (>50% probability)
Likely	4	Will probably occur (21-50% probability)
Possible	3	Might occur under certain conditions (6-20% probability)
Unlikely	2	Not expected but possible (1-5% probability)
Rare	1	Extremely unlikely to occur (<1% probability in 5 years)

Table A1.5. The severity of risk (ρ) levels' definitions.

Level	Score	Description
Catastrophic	5	Devastating health impacts, extreme disruption
Major	4	Severe health impacts, substantial disruption

Moderate	3	Significant health impacts, noticeable disruption
Minor	2	Minor health impacts, limited disruption
Minimal	1	Negligible health impacts, no disruption to daily life

Table A1.6. Risk matrix approach (RMA).

**Risk Scoring**		**Occurrence**				
		1	2	3	4	5
**Severity**	1	1	2	3	4	5
	2	2	4	6	8	10
	3	3	6	9	12	15
	4	4	8	12	16	20
	5	5	10	15	20	25

Table A1.7. The risk ($\rho\iota$) scores' and levels' definitions.

Level	Score	Description
Very High Risk	(20-25)	Emergency response and management system is required
High Risk	(15-19)	Policy is required with urgency to maintain overseeing and enhance governance
Medium Risk	(10-14)	No Policy Needed, however active and close management with guidelines and standards would be enough
Low Risk	(5-9)	No policy needed - Enhanced monitoring can be enough to recover
Very Low Risk	(1-4)	No Policy needed - Routine monitoring

Table A1.8. The strength of supporting evidence (α) levels' definitions.

Level	Score	Description
Very strong	5	Systematic review for randomized control trials (RCTs), meta-analysis, published by Q1 & Q2 journals or referenced to referenced to global organizations *WHO, OECD, CDC, WEF, IMF, etc.
Strong	4	RCTs, other quantitative methodology research with contestant results, results of health economic evaluations (cost effectiveness modeling)
Acceptable	3	Cohort studies, qualitative methodology research results, even published in Q3 & Q4 journals
Considered	2	Non RCTs, Grey literature, white papers, scientific conferences release.
Inconclusive	1	Case studies, mechanism-based reasoning studies or no clear or directly supporting evidence

Table A1.9. The extent of trend or change (γ) levels' definitions.

Level	Score	Description
Embedded widely (Global)	5	Wide exposure/testing (experience) on global level - Perfect alignment with current health priorities and potential to reshape health systems globally.
Strongly tested (Regional)	4	Strong alignment with current health priorities and systems as regional trend whether on the continental or gulf region levels. (responding to regionally emerging change or issue)
Attentively initiated (Federal level)	3	Country (UAE) specific trend that with federal government directives should be discussed, to pioneer.
Early adopting (State level)	2	Emirate (Dubai) specific - Some potential for positive health outcomes in specific contexts based on the directives of the Dubai government, could put Dubai on the early adopter step.
Limited	1	Minimal potential for positive health outcomes, no directives or global impressions.

Table A1.10. The opportunity matrix approach.

Opportunity scoring		Strength of supporting evidence				
		1	2	3	4	5
Extent of change	1	1	2	3	4	5
	2	2	4	6	8	10
	3	3	6	9	12	15
	4	4	8	12	16	20
	5	5	10	15	20	25

Table A1.11. The opportunity in change $(\gamma\alpha)$ levels' definitions.

Level	Score	Description
Exceptional Opportunity	(20-25)	health policy opportunity with exceptional potential impact and very high likelihood of successful implementation. These opportunities represent transformative improvements with overwhelming supporting evidence.
Substantial Opportunity	(15-19)	Health policy opportunity offers significant improvements with strong supporting evidence.
Moderate Opportunity	(10-14)	Health policy represents meaningful improvements with a solid foundation of evidence.
Modest Opportunity	(5-9)	Health policy shows promise for early adoption but requires further development and evidence.
Minimal Opportunity	(1-4)	Health policy represents marginal improvements to existing systems with limited evidence supporting their value

Table A1.12. The potential impact (κ) of implementing the policy - levels' definitions.

Level	Score	Description
Transformative Impact	5	Strong evidence of significant positive impact on outcomes - Affects most or all individuals or processes (76–100%)
High Impact	4	Evidence of moderate to significant positive impact - Affects a large segment of individuals or processes (41–75%)
Moderate Impact	3	Evidence of moderate positive impact - Affects a moderate segment of individuals or processes (16–40%)
Low Impact	2	Limited evidence of positive impact - Affects a small segment of individuals or processes (5–15%)
Minimal Impact	1	Minimal evidence of positive impact - Affects very few individuals or processes (less than 5%)

Table A1.13. The policy development urgency (υ) levels' definitions.

Urgency	Score	Description
Immediate urgency	5	Need to be implemented within 1–3 months
Urgent	4	Need to be finalized and implemented with a timeline of 3–6 months
Necessary	3	As it is included in the annual plan but not a time necessity, has to be finalized and implemented with a timeline of 6–12 months
Next year plan inclusion	2	Not included in the annual plan for this year and has no urgency reasons, can be postponed with implementation timeline above 12 months.
Future or excluded	1	Can be replanned in the future or excluded intervention

Table A1.14. Eisenhower's matrix for priorities.

Priority scoring		Impact				
		1	2	3	4	5
Urgency	1	1	2	3	4	5
	2	2	4	6	8	10

	3	3	6	9	12	15
	4	4	8	12	16	20
	5	5	10	15	20	25

Table A1.15. The priority ($\kappa\upsilon$) scores' and levels' definitions.

Levels	Score	Description
High Priority	(20–25)	Emergency Policy required as soon as possible
Medium High Priority	(15–19)	Urgent Policy is required between 1–3 months
Medium Priority	(10–14)	policy is required between 3–9 months
Medium Low priority	(5–9)	Policy is required to be finalized after 9–12 months
Low priority	(1–4)	Policy is required to be discussed in next year planning activities

Table A1.16. Adjusted Priority Index (API) scores' and levels' definitions.

Index Levels	Score	Description
Crucial	(16.5–25)	Extremely Aligned with Strategy, laws, executive council directive, reactive to high-risk emergencies, affecting broader segment of population or stakeholders, extreme priority to be finalized and implemented as soon as possible (work planned and finalized within first few months)
High Priority	(9.5–16.4)	Highly aligned with strategy and law and directive, however the priority is not high enough (work planned and finalized within 3–6 months)
Medium Priority	(4.5–9.4)	Moderately suiting the strategic goals and objectives with minimal regal requirements, however the priority is medium (work planned and finalized within 6–12 months)
Low Priority	(1.5–4.4)	Limited suitability & alignment with strategy, law and directives with lower priority (work planned and finalized between 12–24months)
Excluded or postponed	(0.1–1.4)	Not aligned or currently suitable to be implemented the priority calculation can be postponed to next year's planning or considered a long-term activity. (work plan to be postponed to future or even excluded)

Testing the model

The initial API score was not treated as a final or self-validating decision; rather, it served as a provisional prioritization signal that was subsequently tested through staged operational reliability tests throughout the policy-development cycle. Risk, opportunity, evidence strength, and problem-definition assumptions are examined in every policy research phase, particularly through situational analysis, current-state assessment, stakeholder evidence review, and benchmarking of regional and international practice. Strategic, legal, and Executive Council alignment scores are re-examined during the final policy approval (by the Dubai Government) phase before publication and launch. Impact scores are to be tested during policy alternatives analysis, including feasibility assessment, simulation of policy benefits, effectiveness appraisal, and economic or risk analysis, where data allows. This staged process allowed the initial API assumptions to be confirmed, refined, or qualified before final policy approval. It provides an embedded operational reliability mechanism within the DHA policy-development process, while recognizing that formal statistical inter-rater reliability testing using independent scorers has not yet been completed and remains a future validation priority.

Bias reduction

Subjective scoring bias was minimized by combining predefined five-point scoring rubrics, evidence-based justification, stakeholder review, and staged verification across the policy-development cycle. Each API score is recorded in an API card with a qualitative rating, numerical score, supporting evidence, and rationale. The initial API score is treated as a provisional prioritization signal: risk, opportunity, and evidence-strength assumptions are tested during situational analysis and external benchmarking; impact assumptions are tested during policy alternatives analysis and simulation where feasible; and strategic, legal, and political alignment scores are re-examined during final policy approval before policy launch. Scoring disagreements are resolved through moderated discussion against the scoring definitions and available evidence. These procedures improve transparency and auditability, while recognizing that expert judgment and institutional context cannot be fully removed from policy prioritization

Table A1.17. Mitigating and safeguarding potential scoring bias

**Identified bias hazards**	**Mitigation mechanisms**	**Applying stage**
Individual scorer judgement	Predefined five-point scoring rubrics	Initial API scoring
Selective interpretation of evidence	Mandatory evidence and rationale field in API card	Initial scoring and review
Overestimation of health risk	Situational analysis, current-state assessment, epidemiological and operational data review	Policy research phase
Overestimation of global opportunity	External benchmarking and review of regional and international policy practice	Benchmarking phase
Overstatement of evidence strength	Evidence hierarchy using CEBM/GRADE-informed principles	Evidence review phase
Strategic or political preference bias	Re-examination during final legal, strategic, and governance approval	Final approval phase
Overstatement of impact	Alternatives analysis, feasibility assessment, benefit simulation, economic or risk analysis where feasible	Policy alternatives and ToC phase
Unresolved disagreement	Moderated discussion using scoring definitions and documented rationale	API review and policy labs
Lack of auditability	API card, decision log, evidence record, and documented rationale	Across all stages

To reduce subjective scoring bias, completion of the API card requires documentation of the qualitative score, numerical score, supporting evidence, and rationale for each criterion. Scores should be reviewed against the predefined scoring rubrics and updated or qualified if subsequent situational analysis, benchmarking, alternatives analysis, or approval-stage review identifies evidence inconsistent with the initial score.

Example of an API card for a policy

Table A1.18. API Card for proposed policy on health data and information security

**Policy Name**	**Health data and information security policy**		
**Responsible organization unit**	Information Security Office – DHA		
**Policy topic/rationale**	Information security aspects of all health information. The policy will address issues related to data and health information security breaches and clarify how to handle health information breaches or cybersecurity attacks. The policy is managed by Informatics and Smart Health and will be updated to fall under the Office of Information Security.		
**Alignment evaluation criteria**	**Qualitative**	**Quantitative**	**Discussion/evidence/rationale**
**Strategic alignment**	Integrated Alignment (initiative)	3	Can be integrated with the initiative for developing policies and legislation for regulating digital health
**Legal alignment**	Complete Alignment	5	Federal law number (2) for 2012 and its amendments on information technology crimes<br>Federal law number (45) for 2021 on protection of personal information
**Alignment with TEC priorities & directives**	Complete Alignment	5	Improving the NABIDH and its applications is one of the TEC priorities for 2025
**Risk of emerging health issue**	Very High Risk	20	One of the defined top technological global risks as per the WEF 2024 Global Risk Report - Ranked No. 5 in UAE as per the same report – Likelihood = 4 & severity = 5
**Opportunity in global trend/change**	Exceptional Opportunity High (Change)	25	health policy opportunity with exceptional potential impact and very high likelihood of successful implementation. These opportunities represent transformative improvements with overwhelming supporting evidence.<br>Evidence strength = 5 & extend of change = 5<br>Evidence 1, Evidence 2, Evidence 3, Evidence 4, Evidence 5, OECD Guidelines,
**Estimated policy impact**	Transformative	5	Affects most or all individuals or processes (76-100%) which is Strong evidence of significant positive impact on outcome
**Policy development necessity (urgency)**	Immediate urgency	5	According to the organization unit request – to be developed before end of 2025
Using qualitative and quantitative assessments to feed all the criteria and using the API formula, the:			
**Adjusted Priority Index (API)**	Crucial	22.3	The topic was classified as Crucial and was therefore included as an accelerated policy research topic in the three-year health policy agenda under the **health data governance policy framework**.

** The API score informed agenda placement, sequencing, and allocation of policy research resources. The score did not determine the final policy solution; policy alternatives and interventions were subsequently assessed through situational analysis, benchmarking, alternatives analysis, theory of change, and final approval*

References

[1] Henderson JC, Venkatraman H. Strategic alignment: leveraging information technology for transforming organizations. *IBM Syst J*. (1993) 32:472–84. doi: 10.1147/sj.382.0472

[2] Kaplan RS, Norton DP. Alignment: Using the Balanced Scorecard to Create Corporate Synergies. Harvard Business Press (2006).

[3] Colombia University, School of Professional Studies. Eisenhower Matrix. https://sps.columbia.edu/sites/default/files/2023-08/Eisenhower%20Matrix.pdf [Accessed September 16, 2025].

[4] Kennedy DR, Porter AL. The illusion of urgency. *Am J Pharm Educ*. (2022) 86: 8914. doi: 10.5688/ajpe8914

[5] Duijm NJ. Recommendations on the use and design of risk matrices. *Safety Sci*. (2015) 76:21–31. doi: 10.1016/j.ssci.2015.02.014

[6] Ni H, Chen A, Chen N. Some extensions on risk matrix approach. *Safety Sci*. (2010) 48:1269–78. doi: 10.1016/j.ssci.2010.04.005

[7] Durieux N, Vandenput S, Pasleau F. Médecine factuelle: la hiérarchisation des preuves par le Centre for Evidence-Based Medicine d'Oxford [OCEBM levels of evidence system]." Rev Med Liège. (2013) 68:644–9. French.

[8] Howick J, Chalmers I, Glasziou P, Greenhalgh T, Heneghan C, Liberati A, et al. The 2011 Oxford CEBM Evidence Levels of Evidence (Introductory Document). Oxford Centre for Evidence-Based Medicine. https://www.cebm.ox.ac.uk/resources/levels-of-evidence/ocebm-levels-of-evidence [Accessed September 16, 2025].

[9] Aguayo-Albasini JL, Flores-Pastor B, Soria-Aledo V. GRADE system: classification of quality of evidence and strength of recommendation. *Cirugía Española* (English Edition). (2014) 92:82–8. doi: 10.1016/j.cireng.2013.08.002

[10] Guyatt GH, Falck-Ytter Y. GRADE: an emerging consensus on rating quality of evidence and strength of recommendations. *BMJ*. (2008) 336:924–6. doi: 10.1136/bmj.39489.470347.AD
