# Supplementary material for: A contemporary approach to developing health policies: the Dubai Health Authority as a case study
Source: Front Public Health. 2026 Jul 1;14:1864765. doi: 10.3389/fpubh.2026.1864765 (PMC13369604; doi:10.3389/fpubh.2026.1864765)
Supplement: Supplementary file 2 [file Data_Sheet_2.pdf]

Appendix 2. Health Policy Frameworks

Background

A health policy framework is an overarching structure that guides the development, implementation, and evaluation of health policies and strategies. The framework usually maintains throughout elements that provide a roadmap for researching policy alternatives, studying the current state, reviewing best practices, and improving health outcomes by integrating various components such as understanding the health system, addressing health determinants, and involving stakeholders. The framework elements vary from the inclusion and exclusion in the policy scope, the policy enablers and the pillars, which represent the challenges, processes and the relevant research topics. Key examples include frameworks for promoting health, which focus on enabling people to control and improve their health, and health systems frameworks that help countries design and govern their overall health systems. [1]

Rationale

Establishing and maintaining a clear policy framework throughout policy development and in policy brief offers several advantages:

- Ensures consistency and efficiency as policy development and management is standardized, ad-hoc processes and confusion are prevented.
- Improves effectiveness as policies are more likely to be adhered to and their intended outcomes achieved if staff understand the process and have confidence that policies are well-developed and authorized.
- Ensures the cohesion of the policy elements and research topics and that all policy interventions are relevant and aligned with the organization's values and high-level strategy.
- Provides a clear view on inclusions and exclusions to the policy study scope and applicability to stakeholders.
- Identifies the policy enablers that support the implementation of any approved intervention.

Illustration method & framework components

- **Policy scope**: A list of all the inclusions and exclusions of policy-relevant topics, issues, data, and processes at the geographic, demographic, stakeholders, and application levels

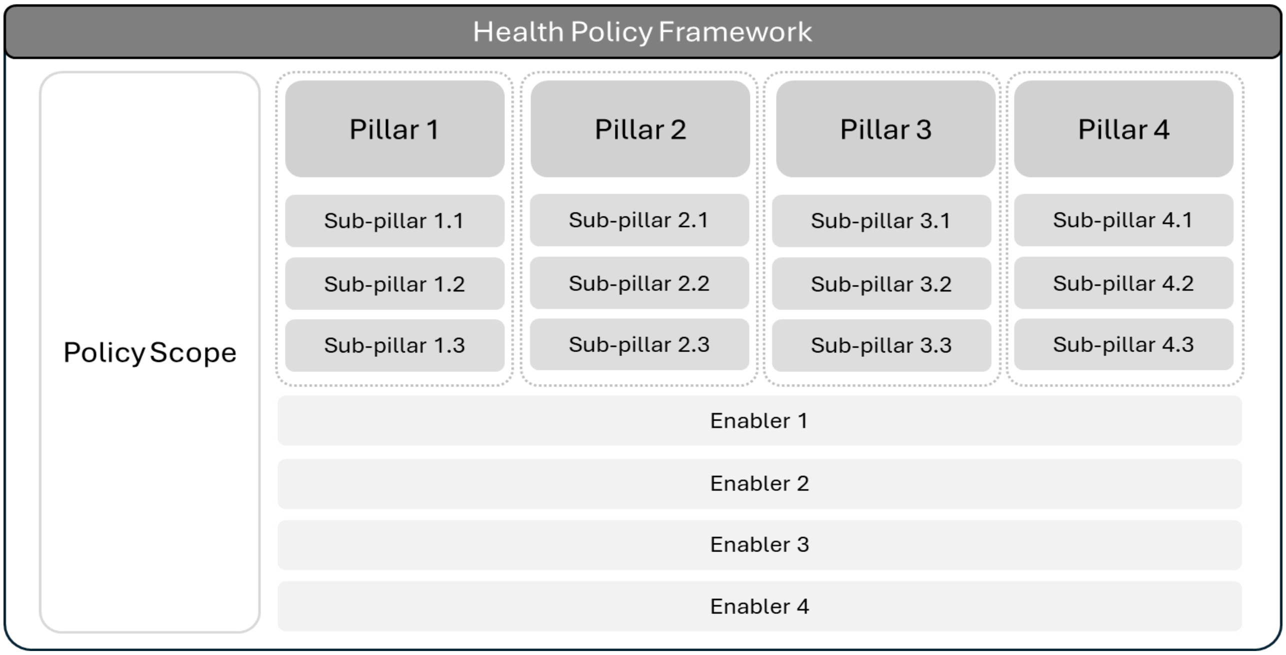

Figure A2.1. Health policy framework illustration tool.

- **Policy framework pillars**: a list of all the main issues, challenges, or primary policy-relevant topics that are included in the policy scope and affect the objectives of the policy, the evaluations, and the prospective impact.
- **Sub-pillars**: the breakdown of the policy framework pillars into research topics for secondary issues of discussion. This list is essential to ensure the scope is observed and the assessment exercises are within the requirements of coverage.
- **Policy enablers**: The policy tools that shall be considered in every pillar assessment and while listing recommendations, policy alternatives, and analyzing the impact of the interventions.

Example of policy framework

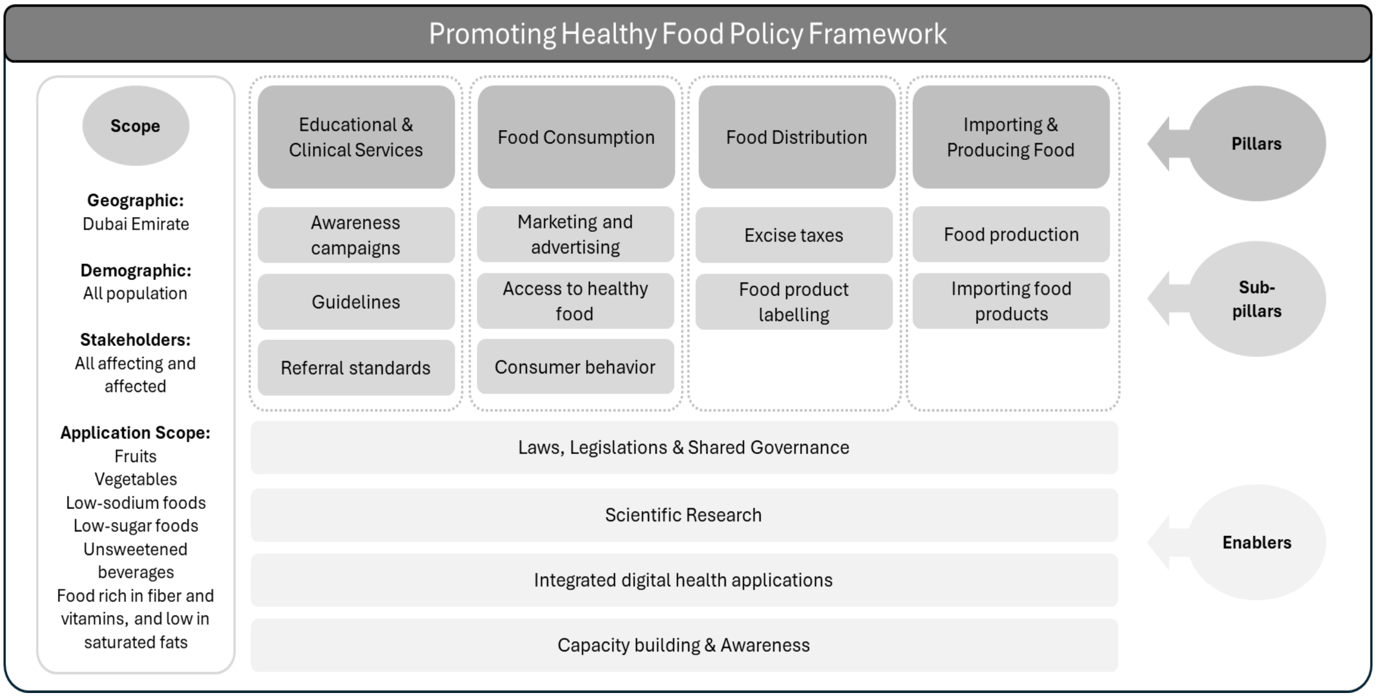

Figure A2.2. The framework for the health policy on "promoting healthy food".

Reference

[1] O'Brien GL Sinnott S-J, Walshe V, Mulcahy M, Byrne S. Health policy triangle framework: narrative review of the recent literature. *Health Policy Open.* (2020) 1:100016. doi: 10.1016/j.hpopen.2020.100016
