# Supplementary material for: A contemporary approach to developing health policies: the Dubai Health Authority as a case study
Source: Front Public Health. 2026 Jul 1;14:1864765. doi: 10.3389/fpubh.2026.1864765 (PMC13369604; doi:10.3389/fpubh.2026.1864765)
Supplement: Supplementary file 3 [file Data_Sheet_3.pdf]

Appendix 3. Stakeholder engagement process

Background

Stakeholder engagement is embedded in the DHA health policy-development methodology to ensure that policy topics, evidence interpretation, recommendations, alternatives, and proposed interventions are informed by the actors who are responsible for, accountable for, affected by, or able to influence the policy area. The process is designed to support transparency, reduce siloed decision-making, and improve the reproducibility of stakeholder selection across policy workstreams. The stakeholder engagement process is linked to the sequential policy labs. It begins during the early policy design phase, when the policy framework is translated into topic-specific activities and the relevant stakeholders are identified. It then continues through stakeholder consultation, policy alternatives analysis, theory-of-change review, and policy launch communication.

Objectives

The stakeholder engagement process has four purposes:

- To identify stakeholders whose mandate, technical expertise, implementation responsibility, or public-facing role makes their involvement necessary.
- To classify stakeholders according to their power and interest in the policy topic.
- To define the appropriate level and timing of engagement for each stakeholder group.
- To create an auditable record of how stakeholder input was obtained, reviewed, incorporated, or escalated.

Method

Stakeholder identification

The process begins in Policy Lab 1 by breaking down the policy framework into its main activities, pillars, sub-pillars, and enablers. For each activity, the policy team lists stakeholders expected to be responsible, accountable, consulted, affected, or informed. Stakeholders may include DHA organizational units, public-sector entities, private healthcare providers, payers, academic or technical experts, professional bodies, civil society organizations, patient or community representatives, and other actors relevant to the policy topic. This exercise led to a discussion at the end of Policy Lab 1, aimed at mapping relationships within the policy topic ecosystem and producing an illustration to support the listing of main activities. (See a mapping example in Figure A3.1). The specific stakeholder list varies by policy workstream. In general, Direct participation of individual patients may not be feasible for every policy topic; where policies directly affect populations, patient or community input is sought through surveys, civil society groups, community representatives, or service-user representatives. If these groups are not included, the reasons should be recorded in the stakeholder register.

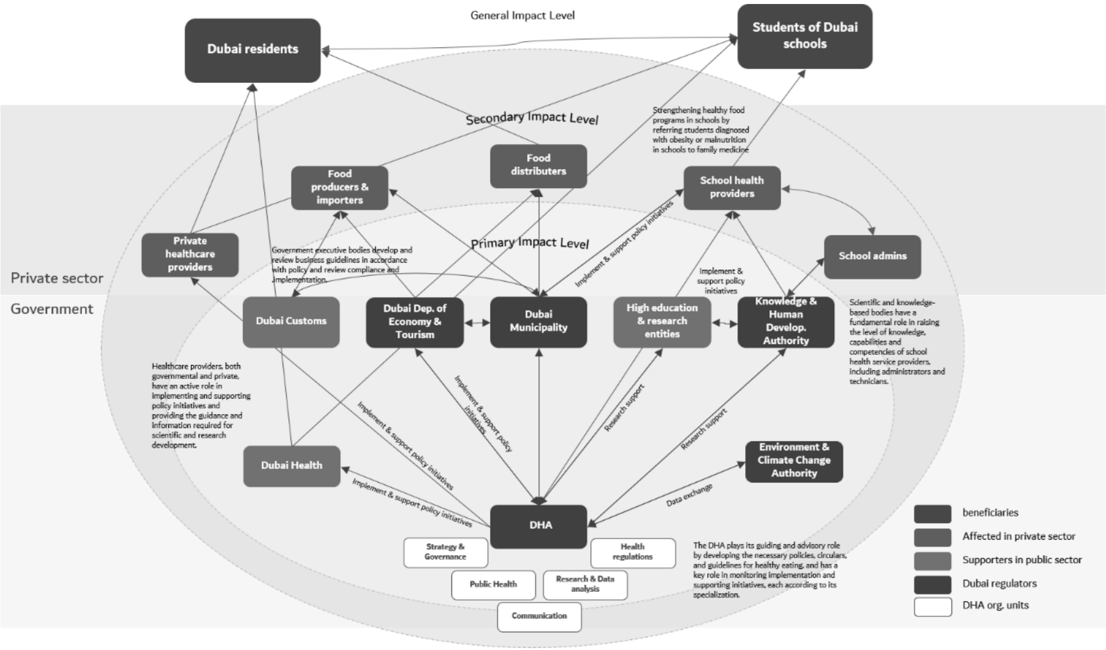

Figure A3.1: Stakeholder Mapping (Example from policy on promoting healthy food)

Power-interest scoring

Stakeholder selection is based on a semi-qualitative power-interest analysis. Power reflects the stakeholder's ability to influence the policy, approve or block implementation, allocate resources, regulate practice, provide data, or affect adoption. Interest reflects the stakeholder's relevance to the policy topic and is estimated by the frequency with which the stakeholder appears across the activity-stakeholder mapping exercise.

During the mapping exercise, relevant internal stakeholders and policy champions review the activity-stakeholder matrix and provide anonymous power scores for each listed stakeholder using the three-point scale shown in Table A3.1. Anonymous scores are averaged at the activity level and then summed across activities to produce a consolidated power score. Interest is calculated by counting how often each stakeholder appears across the policy activities, pillars, sub-pillars, or enablers. The consolidated power score and interest count are then used to generate a stakeholder influence score, which is visualized in a bubble chart (see example in Table A3.2).

Table A3.1: Power scoring definitions

**Power level**	**Score**	**Operational definition**
High power	3	The stakeholder has formal authority, regulatory mandate, approval power, implementation accountability, resource control, or a strong ability to accelerate, block, or materially shape the policy.
Moderate power	2	The stakeholder has relevant technical expertise, data reference, operational responsibility, advisory influence, or partial implementation role, but does not hold final approval or system-level control.
Low power	1	The stakeholder has limited direct authority over the policy but may be affected by the policy, provide contextual insight, or require communication during development or launch.

Table A3.2: Activity stakeholder matrix (Example from stakeholder's analysis made for Promoting health food policy)

**Policy framework pillar**	**Relevant activity** *(example activities)* *Not all actual activities stated for the analysis are mentioned in this example*	**Stakeholder** *(not all stakeholders are mentioned in this example)*													
		**DHA** (Public Health Department)	**DHA** (Health Regulations)	**Dubai Health**	**Dubai Municipality**	**Dubai Department of Economy & Tourism**	**Dubai Customs**	**Ministry of Higher Education & Research**	**Knowledge & Human Develop. Authority**	**Food producers & importers**	**Food distributers**	**School health providers**	**Private healthcare providers**	**School Admins**	**Dubai Population**
Pillar 1 Importing & producing Food	Defining healthy & unhealthy food	3		3	3			2		1					
	Discussing new excise taxes on unhealthy food	2				3	3			1	1				1
	Monitoring the imported food labeling system				1		3								
Pillar 2 Food distribution	Develop enhanced guidelines for standardizing the food labeling system	2			3					1	1				1
	Monitor the implementation of the national food labeling system	1			3										
Pillar 3 Food consumption	Develop standardized regulations for the commercialization of unhealthy food	1	1		3					1	2	1			1
	Monitor the implementation	1	2		3										
Pillar 4 Enhanced medical and education services	Enhance the school health services capacity	3			3				2			2		3	
	Enhance the school's healthy food program	3	3	1	1				2				1	2	1
	Participate in the relevant research work	3		2	1			3	2				2		3
	Standardize the referring protocols to primary healthcare providers	3	3	2									2	1	
	Consolidated power score (summation of power scores)	**22**	**9**	**8**	**21**	**3**	**6**	**5**	**6**	**4**	**4**	**3**	**5**	**6**	**7**
	Consolidated interest score (count of mentions)	**10**	**4**	**4**	**9**	**1**	**2**	**2**	**3**	**4**	**3**	**2**	**3**	**3**	**5**
	Stakeholders' Influence score (power analysis)	**220**	**36**	**32**	**189**	**3**	**12**	**10**	**18**	**16**	**12**	**6**	**15**	**18**	**35**

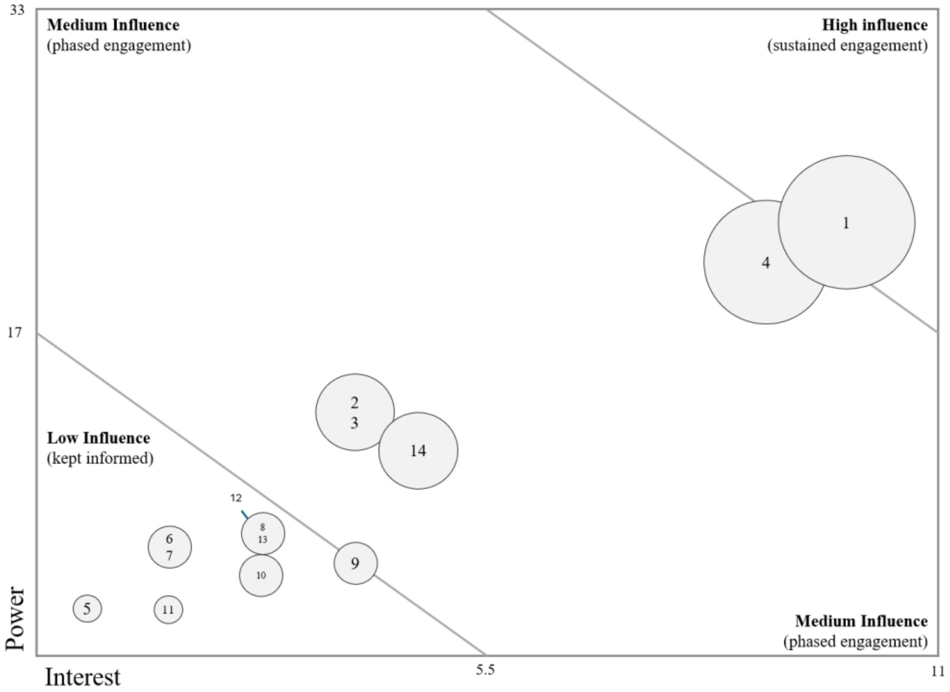

#	*Stakeholder*	*Influence score*
1	DHA (Public Health)	220
2	DHA (Health Regulations)	36
3	Dubai Health	32
4	Dubai Municipality	189
5	Dubai Department of Economy & Tourism	3
6	Dubai Customs	12
7	Ministry of Higher Education & Research	10
8	Knowledge & Human Develop. Authority	18
9	Food producers & importers	16
10	Food distributers	12
11	School health providers	6
12	Private healthcare providers	15
13	School Admins	18
14	Dubai Population	35

Figure A3.2: Semi-qualitative stakeholders' power analysis (based on the example in Table A3.2)

Please note that the scoring values in this example are illustrative and are provided only to demonstrate the stakeholder-analysis method.

Charting stakeholder influence

The final stakeholder influence analysis combines the average power score and the interest score and is illustrated in a bubble chart for easier visualization of the stakeholder power analysis matrix. The power axe reaches its maximum based on the total number of activities multiplied by the highest score of three. Conversely, the interest axe is capped at the total number of activities, representing the maximum contributions. The influence score of each stakeholder determines the size of the bubble; however, the bubble's location on the chart is determined by the intersection of scores on the power and interest axes (see the example in Figure A3.2). Stakeholders with high power and high interest are considered highly influential and invited to participate throughout the policy development process. Stakeholders with moderate influence are invited at defined engagement points, particularly Lab 3 and the final review. Stakeholders with low influence or low direct relevance are kept informed during policy development and at policy launch.

Table A3.3: Stakeholders' engagement levels

**Stakeholder category**	**Selection basis**	**Engagement level**	**Main engagement points**	**Examples**
High-influence stakeholders	High power and high interest	Sustained engagement	Invited to all relevant policy labs, review of evidence, alternatives, ToC, and final policy outputs	The public health department's participation in promoting healthy food policy.
Medium-influence stakeholders	Moderate power or topic relevance	Targeted consultation<br>Selected engagement	Invited mainly to the Lab 3 stakeholder engagement meeting and final review of policy research and ToC	pharmaceutical companies attending the stakeholders' engagement meeting regarding the medication management policy, which is in progress.
Low-influence or information-only stakeholders	Low power or indirect relevance	Kept informed	Updated during development where relevant and informed at policy launch	
Patient, civil society, or community representatives	Included where the policy directly affects service users, communities, or public behavior	Consultation or review, depending on the mapping result	Lab 3, public-facing consultation,<br>Lab 2, feedback through a survey.	- Community representatives (social clubs) participated in the stakeholder engagement meeting in Lab 3 on the smoking policy.

				- The Dubai household survey participated in the situational analysis (Lab 2) of all public health policy topics, such as Smoking, NCDs, and the promotion of healthy food.

Stakeholder engagement roundtables

Aside from the highly influential stakeholders identified in the previous analysis, Lab 3 is the primary structured stakeholder consultation point for policy alternatives. Stakeholders invited to Lab 3 receive a presentation on the policy problem, proposed framework, scope, situational analysis, current-state findings, benchmarking results, and emerging gaps. The purpose is to test the interpretation of the evidence, identify any missing operational or contextual issues, and gather stakeholder views on possible recommendations and policy alternatives. Additionally, various stakeholders can be contacted to share essential data or participate in situational surveys, which is an effective way to collect information and feedback from the population or specific communities.

The Lab 3 policy stakeholder engagement is usually conducted as an extended roundtable(s). Stakeholders may provide feedback during open discussion or through anonymous channels. To reduce dominance by powerful participants and to manage conflict, the discussion may include structured multiple-choice questions, open-ended questions, anonymous digital responses, or written comments. These mechanisms allow stakeholders to express disagreement or propose alternatives without requiring public attribution**.**

Final review and feedback

Before final approval, a version of the policy research output and the proposed theory-of-change solutions is shared with relevant stakeholders for review. This step gives stakeholders an opportunity to identify factual inaccuracies, implementation risks, missing assumptions, or needed revisions. Feedback is consolidated by the policy team, reviewed against the evidence base and policy objectives, and incorporated where appropriate.

Managing disagreement and conflict

Disagreement between stakeholders is expected in policy development and is treated as part of the evidence-generation process. Conflicts are managed through moderated discussion, reference to the policy framework and evidence base, anonymous feedback tools, and documentation of divergent views. Where disagreement cannot be resolved within the stakeholder meeting, the issue is recorded in the stakeholder feedback report and escalated to the policy team, the responsible organizational unit, or decision-makers for review.

Since the policy interventions rely on further benchmarking against best practices, feasibility analysis, benefit analysis, and, when possible, simulated impact, the process does not assume that all stakeholders must agree on every policy alternative. Instead, it requires that disagreements, evidence gaps, and unresolved trade-offs be documented transparently and considered before final policy solutions and theory-of-change proposals are shared with all stakeholders for final review and submission for approval.

Transparency and accountability mechanisms

The stakeholder engagement process is documented in a stakeholder register, an activity-stakeholder matrix, anonymous scoring sheets, averaged power scores, interest counts, an influence map, an invitation list, attendance records, meeting minutes, feedback forms, a consolidated consultation report, a decision log, and a final review record. After Lab 3, a consolidated report is shared with participating stakeholders, summarizing the issues discussed, the feedback received, and the next steps. This improves transparency and allows stakeholders to verify that their input was captured.

The use of predefined scoring definitions, anonymous scoring, averaged results, influence mapping, structured consultation, documented feedback, and final review creates a reproducible process for stakeholder selection and engagement across policy workstreams.

Table A3.4: Reproducibility and transparency mitigation

**Process risk**	**Mitigation mechanisms**
Stakeholder selection based on informal judgment	Use of a structured activity-stakeholder matrix
Overrepresentation of powerful internal voices	Anonymous power scoring and averaging of scores
Missing affected or implementation-relevant stakeholders	Mapping stakeholders against every policy framework activity
Unclear engagement level	Classification into sustained engagement, targeted consultation, or information-only
Dominance or conflict during meetings	Facilitated roundtable, anonymous responses, structured MCQs, and open-ended questions

Loss of stakeholder comments	Consolidated feedback report and decision log
Limited auditability	Stakeholder register, scoring sheets, attendance records, minutes, and final review record
Poor reproducibility across policy topics	Standard scoring definitions and repeatable engagement pathway
