# Supplementary material for: A contemporary approach to developing health policies: the Dubai Health Authority as a case study
Source: Front Public Health. 2026 Jul 1;14:1864765. doi: 10.3389/fpubh.2026.1864765 (PMC13369604; doi:10.3389/fpubh.2026.1864765)
Supplement: Supplementary file 4 [file Data_Sheet_4.pdf]

Appendix 4. Health Policy Theory of Change

Background

Having conducted evidence-based analysis to further define the policy area, its pillars, sub-pillars, gaps, and causes, the last phase of policy development involves defining the policy theory of change (ToC) as a proposal to decision makers, including the intended results of the policy, as measured through impacts, outcomes, and outputs. Policy ToC sets the causal logic of the proposed policy intervention on how the policy is to achieve these four different levels of results. It shifts the focus from the policy inputs and activities to the policy objectives, impacts, outcomes, and outputs.

Rational

A well-structured ToC will:

- Provide a clear understanding of the intended results of the policy, including the planned interventions, impacts, outcomes, and outputs, and their causal links,
- Propose a hypothesis on how change will occur through the policy,
- Provide a clear basis for identifying and appraising options,
- Set clear and quantifiable monitoring and evaluation indicators and targets to measure progress, and
- Provide a powerful communication framework for the different levels of policy results.

Method & ToC Components

Following the formulation of recommendations and the analysis of alternatives, the development of the ToC for the health policy will be contingent upon consensus regarding the proposed policy interventions, anticipated impacts, outcomes, outputs, indicators, and targets. [1] The availability and reliability of data related to the proposed indicators and targets will also play a critical role. This phase should be afforded sufficient time to ensure thorough deliberation, particularly if extensive stakeholder engagement is necessary to achieve alignment across the various levels of the ToC. [2] Furthermore, the selection of policy interventions will require substantial simulation and forecasting efforts. These might include quantitative methodologies such as cost–benefit analysis, cost-effectiveness analysis, economic evaluations, randomized controlled trials (RCTs), and risk assessments, as well as qualitative approaches such as benchmarking successful case studies, analyzing survey data, and using participatory methods such as stakeholder voting.

A good ToC has the following basic components:

- **Suggested policy intervention**: is the starting point for the ToC. It should be mapped into the policy framework pillar and recommendations made based on the gap analysis, which includes the identified issues and causes. Irrelevant policy interventions are a poor example of inconsistent policy mapping and current state assessment. All suggested interventions should be SMART (specific, measurable, achievable, relevant, and time-bounded).
- **Policy intervention inputs** are the direct and indirect resources needed to accomplish the implementation of the suggested intervention. That can include skills, capacity building, structural and infrastructural resources and financial budget. The initial identification of the most relevant stakeholders for implementing such intervention could be a vital point of input.
- **Policy intervention outputs** are the immediate results of implementation. They are the products and/or services that need to be produced or delivered to bring about the identified outcomes. The indicator of output defines the future compliance with policy implementation.
- **Policy intervention outcomes** are the required changes in service users, mostly in terms of capacity and performance that will contribute to achieving the impacts. Outcomes are generally intermediate (short to medium-term). The indicators for the outcome defines the successful implementation for intended short-to-medium term results of the policy.
- **Policy intervention impact** [3] is the change in well-being conditions or widespread societal improvements that need to occur in order to achieve these objectives. Impacts are generally long-term in nature. The impact measures are an essential part of the ToC proposal, which shall include target, baseline, and measuring plans. The impact of a policy intervention could be:
    - **Social**. Social impact relates to the social fabric of the community and the well-being of individuals and families. Well-being includes life satisfaction, relationships, health, education, and community involvement.
    - **Economic**. Economic impact relates to costing activity and growth, public demand, wages, employment, property values, and other macroeconomic indicators.
    - **Environmental**. Environmental impact relates to climate, land, water, air and other natural resources, biodiversity, waste, and so forth.
- **Defined indicators and targets**: The final step in developing the ToC would be to identify indicators relating to impacts, outcomes, and outputs. Indicators are key measures of performance that help to assess the extent to which the desired results have been achieved. Quantifiable targets also need to be set for the various indicators.

- **Policy intervention evidence**, which is the result of the supporting analysis performed before selecting this intervention among the policy alternatives. It can be the results of all the quantitative or qualitative analyses run for the long list of recommendations and alternatives in order to short-list alternative policies. Moreover, it is the overview given to the decision-maker to propose the success of the policy and validity of the prospective impact analysis presented in the ToC.
- **Defined assumptions**: Going through the process of defining the policy interventions, outputs, outcomes, and impacts entails a series of assumptions. Clarifying these assumptions will help identify factors critical to the policy's success. Assumptions could be:
  - causal links between the policy interventions, impacts and outcomes,
  - causal links between the policy outputs and outcomes,
  - contextual or environmental factors that might influence the policy impacts and outcomes.

Example of policy ToC

Table A4.1. Example of a theory of change model for a health policy intervention

**Intervention**	**Input**	**Output**	**Outcome**	**Impact**	**Evidence**	**indicator**	**Target**
Ensure that all employees enjoy a minimum level of health coverage to protect them in case of illness.<br><br>By: Dubai health insurance corporation, health providers and health payers	Study on minimal employee health coverage.<br><br>System of governance<br><br>Agreements with payers and third party	Health insurance coverage for all Dubai residents for emergency and curative healthcare needs	Financial sustainability of the healthcare system<br><br>Reduced government spending<br><br>Increased access to healthcare services<br><br>Enhanced quality of service delivery	Increased labor productivity<br><br>Reduced health risks	Monetized<br><br>Cost–benefit analysis<br><br>Similar country context success stories	% of population covered	% of population covered = 50% in year 2 and 75% in year 3, up from 30% in year 0

References

[1] Mittenthal R. Philanthropy Program Design. International Network on Strategic Philanthropy. (2005) https://nncg.issuelab.org/resources/13925/13925.pdf [Accessed 13 August 13, 2025].

[2] Western and Pacific Child Welfare Implementation Center. Stakeholder Engagement: Tools for Action. (2013) https://www.advancingstates.org/sites/default/files/WPIC_DCFS_Stakeholder_Engagement_Toolkit.pdf [Accessed August 14, 2025].

[3] Khandker SR, Koolwal GB, Samad HA. Handbook on Impact Evaluation: Quantitative Methods and Practices (English). Washington, DC: World Bank (2009). http://documents.worldbank.org/curated/en/650951468335456749 [Accessed August 13, 2025].
